# Sustainable waste management of medical waste in African developing countries: A narrative review

**DOI:** 10.1177/0734242X211029175

**Published:** 2021-07-03

**Authors:** Jade Megan Chisholm, Reza Zamani, Abdelazim M Negm, Noha Said, Mahmoud M Abdel daiem, Mahdieh Dibaj, Mohammad Akrami

**Affiliations:** 1 Medical School, University of Exeter, Exeter, UK; 2 Water and Water Engineering Department, Faculty of Engineering, Zagazig University, Zagazig, Egypt; 3 Environmental Engineering Department, Faculty of Engineering, Zagazig University, Zagazig, Egypt; 4 Civil Engineering Department, College of Engineering, Shaqra University, Al-dawadmi, Ar Riyadh, Saudi Arabia; 5 Department of Engineering, College of Engineering, Mathematics, and Physical Sciences, University of Exeter, Exeter, UK

**Keywords:** Africa, pharmaceutical, waste management, sustainability, developing countries, healthcare waste

## Abstract

Africa is the second populous continent, and its population has the fastest growing rate. Some African countries are still plagued by poverty, poor sanitary conditions and limited resources, such as clean drinking water, food supply, electricity, and effective waste management systems. Underfunded healthcare systems, poor training and lack of awareness of policies and legislations on handling medical waste have led to increased improper handling of waste within hospitals, healthcare facilities and transportation and storage of medical waste. Some countries, including Ethiopia, Botswana, Nigeria and Algeria, do not have national guidelines in place to adhere to the correct disposal of such wastage. Incineration is often the favoured disposal method due to the rapid diminishment of up to 90% of waste, as well as production of heat for boilers or for energy production. This type of method – if not applying the right technologies – potentially creates hazardous risks of its own, such as harmful emissions and residuals. In this study, the sustainability aspects of medical waste management in Africa were reviewed to present resilient solutions for health and environment protection for the next generation in Africa. The findings of this research introduce policies, possible advices and solutions associated with sustainability and medical waste management that can support decision-makers in developing strategies for the sustainability by using the eco-friendly technologies for efficient medical waste treatment and disposal methods and also can serve as a link between the healthcare system, decision-makers, and stakeholders in developing health policies and programmes.

## Introduction

Africa is the second largest continent, with the fastest growing population, and is home to 1.36 billion people. According to the World Bank Group (WBG), 25 out of 54 countries are currently classified as ‘low income’, 19 countries are classified as low-middle income and 10 countries as upper middle income, with only the Seychelles in the ‘high income’ category. Increased population has led to limited resources, leaving a significant number of communities exposed to unhygienic, poor sanitary conditions that are creating a substantial risk for illness and disease (Demirbas, 2011; Kookana et al., 2014). Development in Africa, including agriculture, urbanisation, electrical energy production, waste management, education and infrastructure underpin socio-economic changes across the continent (Omwoma et al., 2017). Changes like these require an enormous workforce, vast resources and extensive planning, as well as effective waste management and monitoring. All of these are in short supply in Africa (Omwoma et al., 2017). Some countries are still plagued and burdened by poverty, resulting in improper burning of all classes of waste with little to no resolution of this issue in sight. The impact of poor waste management has fallen disproportionately on the poverty-stricken communities that have little or no influence on the waste products being illegally dumped near their communities (Du Toit and Bodenstein, 2014). A waste management system consists of appropriate segregation and disposal, with the inclusion of transportation, storage and training facilities for workers. An effective waste management system is often administered by local authorities, with confined capacity for planning, restricted resources, operational monitoring and contract management. These limiting factors make sustainable waste management a difficult proposition (Muluken et al., 2013). The aspect of sustainability and sustainable waste management in this review is the reduction of medical waste that is released into the environment by reducing the volume of waste produced (Demirbas, 2011). Medical waste including pharmaceutical waste that is defined by the World Health Organization (WHO) as ‘expired, unused, spilt and contaminated pharmaceutical products, prescribed and proprietary drugs, vaccines and serum that are no longer required and due to their chemical or biological nature, need to be disposed of carefully’ (Townend, 2001).

The perpetual changes in human behaviour regarding pharmaceuticals have caused elevated rates of pharmaceutical waste in the environment (Ayele and Mamu, 2018). Rapid population growth and urbanisation in low-middle developing income countries (LMDIC) such as many in Africa have led to overconsumption habits and increased wastage levels (Wang et al., 2020). Biennially, an essential medicines list (EML) is released by the WHO. This includes active pharmaceutical ingredients (APIs) that should be used as basic healthcare; however, it does not express specific uses of APIs. Around the globe, beaches and oceans are littered with plastics, offensive waste, clinical healthcare waste and general household waste (Kookana et al., 2014). Various waste disposal methods are used across the continent, with incineration being the favoured method due to quick diminishment of up to 90% of waste, and utilisation of its heat for boilers and in energy production (Ansari et al., 2019). Openly burning these types of waste poses an environmental risk with harmful residuals and emissions (Ansari et al., 2019; Diaz et al., 2005; Omwoma et al., 2017). Medical waste originating from healthcare facilities should be segregated accordingly and treated appropriately (Muluken et al., 2013). An inadequate grasp of medical and other healthcare waste removal policies gives rise to the vulnerability of healthcare personnel and refuse workers as well as the surrounding communities (Giusti, 2009). Limiting factors, such as poor financing, for this sector have resulted in irregular waste collection, improper handling and passing on the responsibility for discarding of the waste on to facility workers (Ruhoy and Daughton, 2008). Waste that is indiscriminately dumped in undesignated places, such as streets and rural areas, is a serious concern for the surrounding residential communities (Dladla et al., 2016). Indiscriminate waste disposal with the addition of improper handling of pharmaceutical waste can lead to APIs posing a threat to land, aquatic environments and humans. If it is left unattended and not stored appropriately, waste can leak out, giving rise to secondary chemical reactions when oxidised and reacting with water and other compounds; therefore, creating a new hazard that had possibly not been envisaged. Other environmental hazards, for instance contamination of surrounding aquatics, and poisonous emissions from open burning of medical waste must be considered (Dladla et al., 2016). Indiscriminate waste disposal also raises the opportunity that medicines will be returned to the community, as they may be able to profit from unused or partially used prescription medicines, including painkillers (Ruhoy and Daughton, 2008).

Throughout the countries of Africa, including Algeria, Nigeria, Ethiopia, Botswana, Ghana and South Africa, state and private hospitals have not shown a significant difference in the way medical waste is managed, with studies suggesting some healthcare workers and waste officials were not aware of policies surrounding the handling of waste nor of its final disposal method (Oli et al., 2016). Regardless of how the institutions are funded, not all hospitals and other healthcare centres have colour-coded bags for classified waste such as pharmaceutical, radioactive and clinical, thus rendering waste segregation impractical (Ruhoy and Daughton, 2008). Contamination of regular waste from the place of origin could create secondary chemical reactions that would cause a wider hazardous issue for the population. Pharmacies in countries, such as Algeria, Botswana, Ethiopia and South Africa are not acquainted with how or where the medical waste is disposed of, and limited funding does not allow for contractual services, for example removal of medical waste, transportation and storage. This is left for the workers of the facilities in each organisation to address. Medical waste will not only debilitate the communities by hazardous waste accumulation on the land, in aquatic and aerial environments, including the presence of APIs, but will also affect the health and well-being of the entire population and national economy. If healthcare waste is segregated adequately, general waste can be harvested for biofuels, leading the national economy in a positive direction and giving plentiful opportunities for sustainability. A proposed solution to create a reduce–reuse–recycle system can reduce medical waste and drastically change the outlook of medical waste handling while at the same adding a revenue stream (Liu and Yao, 2018).

In contrast, other continents around the world, including Europe and North America, have diverse practices for the disposal of classified waste. In the late 20th century to early 21st century, steel and iron industries would deposit chemical toxic waste into the rivers of the UK and Germany, this has led to the accumulation of high levels of coal, nitrate and iron (Aziale and Asafo-Adjei, 2013). This has caused devastating effects on wildlife and water quality throughout. Developed countries, such as the UK, Ireland and USA, depend heavily on landfill waste to reduce waste accumulation and use recycling wherever possible (Giusti, 2009). Continuous ways of developing eco-friendly products are essential to form a circular economy. Landfill, although a sufficient complex of large quantities of waste, is now deemed a source of polluted ground water, therefore increasing the economic issue (Aziale and Asafo-Adjei, 2013; Dioha and Kumar, 2020). Human behaviour plays a critical role in waste management. All policies and guidelines set by governments and official standards for proper handling of waste should be adhered to across the world. Unfortunately, many LMDICs are so exhausted of finances and burdened by effective climate changes that they cannot fulfil the requirements. It is then left to the local authorities to stretch resources, which in turn leaves many rural areas without. It is vital to understand the importance of medical waste, the impact this waste has on the environment and the secondary effects should medical waste be left in inappropriate conditions. A more substantial waste management model should be implemented to allow countries to thrive and take control of their waste. Table 1 shows the types of waste disposal technology, the country in which they are active and an alternative solution to the current methods. It is crucial to enforce new innovative solutions to current disposal methods, to advance waste management globally, and for countries to be responsible for waste in their own environment as opposed to shipping off excess waste to an LMIC to reduce costs, and to allow LMICs to take control of their waste. Simple measures, such as designated recycling areas, recycling centres and education on composting in rural areas, would be beneficial to many households across Africa. The bustling cities and rural areas of Africa have embraced socio-economic changes; however, the continent is still affected by severe poverty, natural disasters and limited resources. The extensive construction of built communities has devastatingly affected natural resources (Omwoma et al., 2017). These challenges are a widespread concern. Subsequently, many families are reliant on the income from working in current waste management conditions and in large quantities, and the effect of this on local economies would need to be considered (Dioha and Kumar, 2020). Additionally, in the countries of Africa (despite some remaining involved in conflict) where there is a possibility to be unanimous, through education, finances and democracy, there is a possibility for a bright and greener future. A lack of these sectors has brought deprivation and harsh consequences for many communities across the continent.

**Table 1.** Current disposal methods, their risk and solutions in countries.

Disposal method	Place/country of the study	Identified risks	Recommended solutions	References and year of publication for the study
Incineration	Nigeria, Algeria, Botswana, Ethiopia and South Africa	Airborne contamination	Harnessing energy from incineration, appropriate incinerators that are serviced to ensure safety	Mmereki et al. (2017)
				Awodele et al. (2016); Mmereki et al. (2017)
				Tadesse and Kumie (2014)
				Bendjoudi et al. (2009)
Open dumping	South Africa, Nigeria and Botswana	Land contamination/risk of contact in the surrounding communities	Prohibition of illegal dumping	Awodele et al. (2016)
			Designated sites for waste	Bendjoudi et al. (2009)
Landfill	South Africa, Nigeria and Botswana	Land contamination, risk of contact in the surrounding communities/overflow into oceans	Recycle, general waste segregation for biofuels	Awodele et al. (2016); Mmereki et al. (2017)
		Lasting waste in the environment		Bendjoudi et al. (2009)
Autoclaving	South Africa and Nigeria	Expenses for electrical usage	Solar energy/wind	Awodele et al. (2016)
				Bendjoudi et al. (2009)
Chemical disinfecting	Algeria	Risk of contact with chemicals, possibility of not treating all pathogenic substances leading to contamination after treatment	Neutralisation or distillation of ethanol	Bendjoudi et al. (2009)
			Using chemicals that are not harmful to humans and animals and do not disturb water quality	
			The use of chemicals that appropriately destroy all pathogenic substances	
Indiscriminate waste	Algeria	Build-up of solid waste in the communities, APIs in the environment	Designated recycling centres, available DUMP programmes for pharmaceutical waste, education on recycling, prohibition of illegal dumping	Bendjoudi et al. (2009)
		Education on reduce, reuse, recycle		

DUMP: disposal of unused medicines programme.

From this back ground, the main objective of this study is reviewing the sustainability aspects of medical waste management in developing countries of Africa to present resilient solutions for health and environment protection for the next generation in Africa. This objective can be achieved by studying the current situation of healthcare system in the different countries, identifying the challenges to governments, finding the approaches and suggestions to overcome these challenges, strategies and action plans should be undertaken and finally presenting the policies, possible advices and solutions associated with sustainability and medical waste management that can support decision-makers in developing strategies for the sustainability by using the eco-friendly technologies for efficient medical waste treatment and disposal methods and can serve as a link between the healthcare system, decision-makers and stakeholders in developing health policies and programmes.

## Medical waste

In recent years, the generation of medical wastes has increased significantly due to the increase in population, facilities of healthcare and hospital number and medicine product wastes, especially after spreading coronavirus (COVID-19) pandemic (Arab et al., 2008; Taghipour and Mosaferi, 2009). Many countries recorded a high increase in their wastes; five times higher than the waste amount before spreading the pandemic (WHO, 2020). According to WHO, about 75%–90% of the wastes generated from different healthcare facilities can be considered as non-hazardous, whereas 10%–25% are hazardous wastes (Chartier et al., 2014). Around 0.5 kg (bed-day)^−1^ of hazardous waste are generated in the high-income countries, whereas 0.2 kg (bed-day)^−1^ are generated in low-income countries (Ali et al., 2017). The hazardous wastes include infectious, radioactive, toxic or genotoxic items that can cause environmental and occupational health risks (Ali et al., 2017). Furthermore, improper disposal of medical wastes into garbage bins can expose children, animals and garbage collectors to serious health hazards (Sefouhi et al., 2013). Moreover, toxic medication wastes can enter in food chains and biological systems of human. This can lead to chronic and acute toxic effects among humans and can affect useful microorganisms, insects, animals and plants (Bataduwaarachchi et al., 2018).

In England, the National Health Service (NHS) have issued a ‘Principles on the Disposal of Waste Pharmaceuticals used within Community Health Services’ document providing the correct guidance on how to dispose of pharmaceutical waste. It states how pharmaceutical waste should be segregated into either cytotoxic and cytostatic medicine, or other medicines. This is further divided into four sub-groups, cytotoxic and cytostatic; medicines that are pharmaceutically non-hazardous (meaning non-cytotoxic and non-cytostatic), not pharmaceutically active, or possessing no hazardous properties, such as glucose or saline; medicines that are flammable, harmful, irritant, oxidising or eco-toxic. The policy states how to correctly colour code boxes and seal them once full, and that a transfer note should be assigned. This is an example of efficient, responsible and safe pharmaceutical waste management, allowing reduced impacts of toxic chemicals or harmful exposure to the environment in England.

Over the years, the environment has become a public concern due to pollution-related illnesses. Many workers have suffered chronic illnesses related to long-term low concentrations of harmful substances. Others have suffered from illnesses related to short-term high concentrations of harmful chemicals or substances. Any personnel in the presence of waste that causes harmful by-products will always be at risk, even with the best technologies available (Giusti, 2009). Substandard incinerators will result in incomplete burning of toxic organic substances, releasing harmful emissions into the air and surrounding work areas, which in turn could affect staff, particularly if they are not properly equipped with protective kits (Olaniyi et al., 2018).

### Medical waste management in Africa

**Figure 1.**
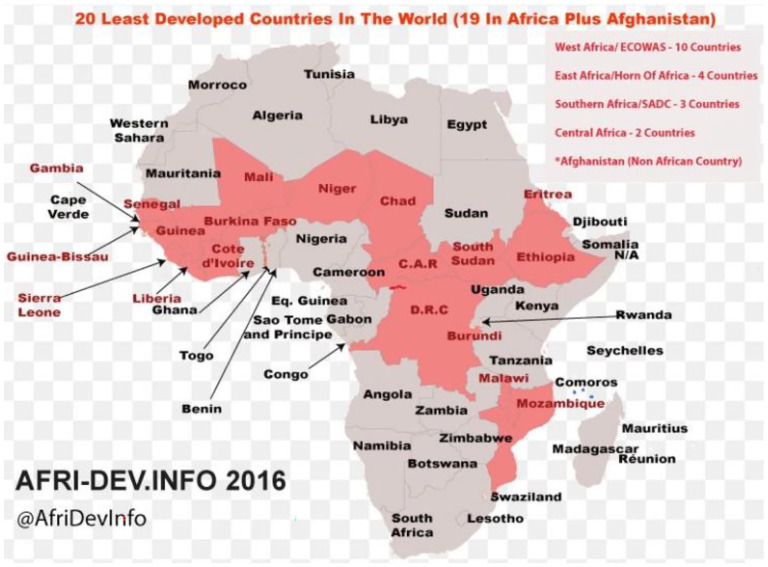
Map of Africa showing developed and developing countries (AFRI-DEV.INFO, 2016).

Africa is estimated to have 67,740 health facilities and produce approximately 282,447 tonnes of medical waste every year (Udofia et al., 2015). Figure 1 illustrates map of Africa showing developed and developing countries. Most of the African countries lack legislation for medical waste management. For example, Eritrea, Lesotho and Ghana have no legislation for healthcare waste management, whereas Kenya, Nigeria, and Gambia are signatories to the Stockholm Convention with few relevant laws (Zafar, 2019). The lack of sanitary landfills has led to the increased use of crudely designed incinerators. Gambia, Ghana, Lesotho, Nigeria, Senegal and Tanzania have no sanitary landfills, whereas Kenya and Zambia only have crude dumpsites (Udofia et al., 2015). Regular disposal of pharmaceutical waste into ‘general waste’ has allowed streams of different medicines to enter landfill and aquatic environments in Africa. This has affected the quality of the surrounding land and water accessed by the residents and wildlife (Lexchin, 2018). This is especially true for the developing countries, which are more vulnerable to pharmaceutical waste threats (Ansari et al., 2019).

The Department of Environmental Affairs (DEA) of South Africa has been on the forefront of drafting waste management guidelines in South Africa, as well as monitoring and conducting relevant studies on the subject nationwide. However, Maseko (2014) identified gaps in policy framework nationally and institutionally on medical waste management, and there were poor waste management practices due to poor training, inadequate infrastructure and resources as well as poor budget support (Maseko, 2014). Otherwise, the Health Professional Council of South Africa (HPCSA) developed a comprehensive healthcare risk waste (HCRW) management guideline booklet that provided a full definition of HCRW and classified the waste into the various sub-categories. It also discusses the risks of mismanagement of the waste to the society and highlighted the roles and responsibilities of each health worker in proper HCRW management (Olaniyi et al., 2019).

In Ethiopia and Kenya, there is an absence of a national policy aimed at the control of the safe disposal of unused medicines; however, Ghana has implemented a disposal of unused medicine programme (DUMP). The introduction of effective programmes, such as DUMP, has allowed for adequate management of pharmaceutical waste that has contributed to a significant reduction of waste in the environment (Dioha and Kumar, 2020). Some of the countries of Africa have stretched finances and are unable to accommodate proper disposal methods and have resorted to other means of disposal (Betey and Essel, 2013). In 1999, the WHO developed a worldwide guide for the disposal of unwanted pharmaceutical waste during and after emergencies. Due to natural disasters and conflicts between countries, humanitarian efforts meant that large quantities of unwanted pharmaceuticals arrived past or near their expiration. Furthermore, the introduction of this documentation developed by the WHO meant that unused pharmaceutical waste could be sufficiently dealt with and allowed countries to dispose of their waste appropriately and efficiently. Diaz et al. (2005) had discovered that in 1996, Botswana implemented a code of practice for medical waste, but studies had suggested that healthcare workers in Botswana were unaware that this documentation had been enforced (Windfeld and Brooks, 2015).

In South Africa today, medical waste and more diverse medical waste is defined and legislated through a variety of policies and legal documentation and is also assessed based on the risk it may cause to health (Hodes, 2019). South Africa’s Department of Health issued a policy on medical waste and included a step-by-step guide on the correct management of medical waste, including storage and contacting the contractors for removal. In addition, the policy has stated the next steps if a contractor is unavailable for the removal, and it is apparent that a pharmacist must be present in addition to a member of the pharmacy personnel. The waste from wards or hospitals must be sent back to the corresponding pharmacy, and the policy implemented must be followed once it has reached the pharmacist. Green specibins are provided by the waste company, and waste must be recorded to allow the pharmacist and contractors to ascertain the contents of the bins. Green specibins can also be acquired through the WHO. Furthermore, the policy and procedures for medical waste state that this does not cover radioactive medical waste, and therefore a different method is followed. This policy should be directly followed by all medical personnel and waste contractors. No medical waste should be stored for more than 90 days from the day it was sealed to the date of disposal. These outlined documentations are official and should be used as templates for all countries to consider if they do not already have policies in place.

In South Africa, incineration is used for many sources, such as heat for boilers and separation of metals that can be reused (Ansari et al., 2019). Not all reach the required temperature to burn healthcare waste: the range of incinerators is 600°C–1000°C. In addition, solid waste management reviews are often neglected due to finances being diverted to other services in the healthcare sector. Although policies and legislation remain in place along with emerging practices, there are no sustainable trends concerning the discarding of classified healthcare waste in most developing nations (Bazmi et al., 2011; Windfeld and Brooks, 2015). For example, a hospital in the city of Batna, Algeria, generates what it is described as risky healthcare waste (RHW) of 1.72 kg (bed-day)^−1^, contributing to a total of 1114 kg of RHW per day for this hospital (Ansari et al., 2019; Sefouhi et al., 2013). The national average in Algeria for RHW is 0.72 kg (bed-day)^−1^ (Sefouhi et al., 2013). Further study has revealed that the highest generation rate of solid healthcare waste belonged to Ethiopia at 6.03 kg (bed-day)^−1^ (Ansari et al., 2019). In addition, a study that reviewed seven hospitals in Lagos, Nigeria found that the Lagos waste management authority (LWMA) had introduced intervention programmes to ensure conformity and safe waste management through its processes. This study also suggested that only one hospital had an incinerator to dispose of sharps only, and the LWMA uses hydroclave technology to treat its waste. This same study concluded that there were no policies or guidelines in any of the seven hospitals assessed for treating healthcare waste, although it found that all seven hospitals generally segregated their general and medical waste, with healthcare workers having access to personal protective equipment (PPE). Private hospitals and state hospitals are not significantly different with regard to waste management. Many healthcare facility workers are poorly trained on policies for discarding different classifications of waste. Multiple studies have suggested change has been implemented in Nigeria, and more healthcare workers in the public sector have attended waste management training sessions on how to correctly handle healthcare waste than have done in the private sector, and they were more aware of the waste management scheme. Furthermore, a waste management committee was established but does not exist in the public sector, and only a few have been constructed in private hospitals and healthcare facilities (Oli et al., 2016).

In Egypt, the issue of hazardous medical wastes management has acquired an increasing interest in the last two decades, as the awareness of their serious health effects has increased on both public and governmental levels (Shouman et al., 2013). According to WHO, the average amount of waste per bed per day is estimated to range from 0.7 to 1.7 kg bed^−1^ in Egypt. Data obtained from the Ministry of Health in Egypt shows that the proportion of hazardous medical waste is in the range of 25%–30% (Aboelnour and Abuelela, 2019). This big percentage may be due to inappropriate healthcare waste segregation. In Egypt, the technologies applied for medical waste treatment are incineration, steam sterilisation and chemical sterilisation. Incineration represents the most common method applied in Egypt (Shouman et al., 2013). Contaminated environments increase the risk of transmission of healthcare-associated infections. One study showed the increase adherence to waste management policy at healthcare facility in Egypt (Oli et al., 2016). That study clearly demonstrated the high risk of hepatitis infection among workers handling, especially medical waste (El-Gilany et al., 2013). Therefore, safety measures, including appropriate PPE, must be available for those workers in addition to educational and training programmes in order to do their job in a safe working environment.

In Morocco, there is no information at present regarding the quantities of all types of medical waste actually collected and treated in the country. Moreover, little information is available regarding generation handling and disposal of waste (Mbarki et al., 2013). The annual production of medical waste from the 142 public hospitals is estimated at 21,000 tonnes year^−1^, including 6000 tonnes of infectious medical waste. Medical waste processing still requires improvement. Even though many hospitals are equipped with an incinerator, most of these do not work or are obsolete. It is estimated that most of this waste is stored in public landfills, which is a serious problem, since it is not only a source of environmental pollution but also a potential source for the spread of infectious diseases (Asfaw, 2014).

Nigeria at present does not have a coordinated healthcare waste management system, especially in the area of segregation, collection, storage, treatment and disposal (Uchechukwu et al., 2017). The level of awareness among healthcare workers regarding healthcare waste has not been adequately documented in Nigeria (Çinar et al., 1998). Many health facilities in Nigeria do not have a specific policy for their waste management (Awodele et al., 2016). In Cameroon, medical waste management is a serious concern, and therefore there is need for an evaluation of current management practices to enable planning for better waste management (Dzekashu et al., 2017).

In Tunisia, the current quantity of medical waste is estimated at 18,000 tonnes year^−1^, including 8000 tonnes of hazardous waste. The separation of waste tends to not be practiced, and hazardous waste is mixed with general municipal waste, ending up in open-field dumps. Spontaneous combustion and inadequate incineration practices are responsible for 90% of the total amount of dioxins and furans emitted in the country (Çinar et al., 1998). In Libya, little information is available regarding generation, handling and disposal of medical waste. This fact hinders the development and implementation of hospital waste management schemes. Sawalem et al. (2009) found that the average waste generation rate was found to be 1.3 kg (patient-day) ^−1^, comprised of 72% general healthcare waste (non-risk) and 28% hazardous waste. Furthermore, the hospitals had neither guideline for separated collection and classification nor methods for storage and disposal of generated waste. This deficiency indicates the need for an adequate medical waste management strategy to improve and control the existing situation. In Sudan, the estimated average generated rate of healthcare wastes ranged from 0.38 to 0.87 kg (bed-day) ^−1^. The study of Taghipour and Mosaferi showed that the management of healthcare wastes is inefficient, as all wastes are mixed with domestic wastes and disposed of improperly. The study attributed this to many reasons, including lack of waste segregation at the source, lack of policies, failure of planning, inadequate training, lack of awareness of the hazardous nature of such kinds of waste, weak infrastructure and a lack of suitable treatment technologies. Table 1 outlines the methods used in the countries of Africa to dispose of hazardous healthcare waste and the risks associated with these. Based on the outcome of the researched papers, a solutions column has proposed alternatives to the current technologies for the reduction of emissions and risks to workers.

## Methods

Africa was chosen for this review due to the consistent studies that show both ineffective and effective waste management policies across the continent. The WBG website was searched to establish the financial position of each African country according to the Gross domestic product (GDP). This review used data from Science Direct and Web of Science database during the year 2020 to explore research published in journals, book chapters and studies on clinical healthcare waste and medical waste in Africa. The main keywords to search the database included healthcare waste, hospital waste, medical waste, infectious waste, clinical waste, sustainable waste management in developing countries in Africa, etc. The papers included in this review include those published between the years 1998 and 2020. This was done to identify current situation and disposal methods for medical wastes as well as to investigate the possible solutions for sustainable waste management in developing countries of Africa. Initial search yielded more than 1000 research articles in English language. These articles were analysed individually to assess if they focused on medical waste management (waste collection, segregation, transportation, storage and disposal practices in Africa developing countries). Thus, general and non-relevant articles were excluded from these articles. At the end, a total of 81 research items has been retained including 76 research articles, two MSc theses, two technical reports and one book chapter. The selected publications reported findings including healthcare facilities in around 18 different African countries.

## Results

### Current situation in developing countries

The level of healthcare waste management can be used as a quality indicator of the healthcare system of the country or the institution (Bataduwaarachchi et al., 2018). Many developed countries apply strict guidelines for medical waste management chains including segregation, storage and transportation (Ali et al., 2017; Jang et al., 2006; Marinković et al., 2008). On the other hand, constrained resources and lack in established programmes in the developing countries are obstacles to achieve effective healthcare waste management (Caniato et al., 2015). Some cases in previous studies have been detected in the developing countries including the following: hospitals lack in properly labelled waste containers and safe storage rooms (Yong et al., 2009); poor condition of the containers and lack of disinfection (Bazrafshan and Mostafapoor, 2011); the store rooms are used to store other items, such as the cleaning equipment (Da Silva et al., 2005); waste is stored in open dumps in the vicinity of the hospital (Manga et al., 2011); the containers are without lids and are not emptied until they are completely full, which can result in onsite waste spillage, lack of PPE for waste transporters (Abd El-Salam, 2010), lack of proper trolleys and transportation in unsuitable vehicles passing through residential areas can cause leakages and accidents (Sawalem et al., 2009); autoclave and disinfection of wastes before disposal is limited to a few hospitals (Manga et al., 2011); thrown of wastes on the road sides and burning of wastes in open landfill sites, which can cause environmental pollution (Mbongwe et al., 2008).

Furthermore, the disposal of medical wastes has been found in different developing countries include the following: open dumping or burning of wastes, although it is cheap and easily available, reduce waste volume and avoid its spreading, but it exposes the public to health risks through direct contact or indirectly by land and water pollution as well as air pollution due to release toxic gasses into the atmosphere during the process (Nemathaga et al., 2008). Incineration technique is used for disposal of pathological wastes, sharps and other medical wastes, which cannot be reused, recycled or disposed of in a landfill (Hossain et al., 2011). However, many incinerators are made locally and designed poorly, and use fossil fuel as coal for burning process, so they are not able to achieve a complete waste combustion and result in a huge amount of ash (Nemathaga et al., 2008). Moreover, the waste residues and ashes are finally disposed of at landfill sites (Bataduwaarachchi et al., 2018). Autoclaving is alternative method for waste treatment and cheaper than incineration. Even though it is helping to get rid of the bacteria from sharps and medical wastes that contaminated with blood and human secretions, but the autoclaved wastes should be retreated with other methods before their final disposal (Jang et al., 2006). In addition, the high quantity of wastes is difficult to be autoclaved because it needs high length of time to achieve the optimum temperature of autoclaving (Nemathaga et al., 2008). Microwave disinfection of wastes is a modification of autoclaving method. However, wastes that contained metal objects cannot be microwaved to avoid the generation of dangerous sparks (Windfeld and Brooks, 2015). Landfills in developing countries are not properly constructed and operated like open dumping and the dumped wastes are burned later (Nemathaga et al., 2008). The wastes can discharge into ground and surface water causing water contamination (Bataduwaarachchi et al., 2018).

### Challenges to governments

Challenging to governments in developing countries could include the following: the political instability, trade-union pressure and pressure from multinational medical companies, lack of necessary laws, regulations and clear policy; lack of funds and insufficient man power, further waste of funds and manpower; lack of efficiency in the government sector and the difficulty of accessing rural population to municipals; lack the technology and the skills required to implement and monitor medical waste management programmes (Bataduwaarachchi et al., 2018); finally the need to increase the capacity to handle and treat wastes without delay, especially after spreading COVID-19 pandemic and the high increase of the wastes (Ali et al., 2017; WHO, 2020). Considering these unique challenges, abrupt transition towards an advanced system may be difficult in developing countries.

### Related research studies

The economic and environmental performance index of hospitals’ solid waste were evaluated for the developing countries (Ansari et al., 2019). This review study summarised the education of healthcare workers with regard to the risks of solid healthcare waste management and observed the hospitals’ solid waste generation rate, its composition, GDP per capita and the environmental performance index for the reported developing countries. In the city of Harar, Eastern Ethiopia, the required knowledge, attitude and practice towards disposal of unused and expired medicals among a community was analysed by Ayele and Mamu (2018). This study was conducted to identify accumulated medicals in 695 households including knowledge of medical waste disposal, and the impact on the environment of its improper disposal. This study also suggested the importance and urgency of guidelines for the correct disposal of medical waste for the public. Alternative solutions for the treatment and disposal of healthcare wastes in developing countries were presented by examining the types of technology and the properties for the disposal of healthcare waste (Diaz et al., 2005). Different disposal methods were reported in detail in this study as well as an indication of what waste should be treated by each technology. This paper included an extensive description of chemical disinfection, including types of alcohol and other substances that can be used and their characteristics.

A review of factors associated with indiscriminate dumping of waste in 11 African countries was the topic of another study. This review discussed the impact of indiscriminate dumping of all types of waste in 11 African countries. The author examined how waste dumping in undesignated areas has extensively impacted the surrounding environment, water facilities and communities (Dladla et al., 2016). Furthermore, the paper discussed the importance of regulating this. Demirbas (2011) considered all aspects of how a waste management system should be implemented. This paper also has discussed biomass and bio-waste resources, as well as waste classifications and waste management, including transportation, storage and proper handling of healthcare waste.

Sustainable energy transitions in sub-Saharan Africa’s residential sector, in the case of Nigeria, were reviewed by Dioha and Kumar (2020). This study presented the effective and sustainable energy system models for Nigeria and the impact of a new residential energy model for the improvement of current fossil fuel derived energy. Another review of waste management practices and their impact on human health examined the most recent policies and information on waste disposal methods in the world, including the European Union (EU), Organisation for Economic Co-operation and Development (OECD) and developing countries (Giusti, 2009). It also included analysis on pollution-related illnesses and the impact of these on residents living near landfill sites, incinerators, composting facilities and nuclear plants. Pharmatrash in South Africa had identified the outcome of medical waste after it has left the place of origin, including purchased, consumed and discarded medical waste. It also recognised the abuse of analgesics in South Africa, with the primary focus on codeine (Hodes, 2019). Potential ecological footprints of active medical ingredients focused on the API consumption in humans in the environment, assessing the differences between the low–middle–high income countries. In addition, Kookana et al. (2014) discussed the sections of population, manufacture, prescription and demographics as well as the treatment, disposal and reuse of waste and wastewater. For Ghana, a study was conducted to better understand the challenges of waste management companies’ facilities in executing a clean city (Oteng-Ababio, 2012). This study also examined inadequate waste management in households across the Metropolis of Ghana, including bin collection and waste segregation.

The major assessment of medical accumulation, with the inclusion of why and where medicines accumulate was identified by Ruhoy and Daughton (2008) . The primary topics discussed were locations where drugs are used and the diversity of their location, pollution prevention and reduction technologies. They criticised the processes, actions and behaviour that fuel the consumption and accumulation and disposal of medicals with the inclusion of active medical ingredients (APIs) and the substantial impacts on the surroundings. Muluken et al. (2013) conducted a study of 260 workers with a structured questionnaire and an observational checklist over the month of April to May 2011 to collect data on the current knowledge of healthcare workers regarding healthcare waste policies and legislation, disease transmission among healthcare waste and segregation of waste and training. This study helps in investigating the purpose of healthcare waste management among healthcare workers in the town of Gondar, Ethiopia. The management of healthcare waste in a hospital in the city of Batna, Algeria was assessed and critically reviewed by Sefouhi et al. (2013). This paper also identified risk management, including the identification, prioritisation and assessments of risks. Also inspected was the governance from the authorities in Algeria to improve the waste management systems in healthcare facilities and hospital settings. A new medical waste disposal method was proposed by Du Toit and Bodenstein (2014), who observed a legal perspective surrounding the legislation on dumping of medical waste in South Africa. The article also discussed good medical practice and standard operations for disposal (Du Toit and Bodenstein, 2014). The most common sources of medical waste and the appropriate legislation and governance were reviewed by Windfeld and Brooks (2015). The handling and disposal methods were identified and included the issue concerning the unclear guidance on what waste should be defined as ‘infectious’ and unnecessary classification of non-infectious waste causing an increase in disposal costs. Awodele et al. (2016) assessed the medical waste management in hospitals in Nigeria by investigating seven hospitals in Lagos, Nigeria and provided a discussion of how the LWMA has impacted medical waste management and the management of the healthcare waste of all of the seven hospitals including policies, treatment, storage facilities and transportation, and how the healthcare workers address these processes (Awodele et al., 2016).

### Current treatment and disposal methods

It is apparent that a sustainable option for medical waste in Africa would be recycling non-hazardous healthcare waste for energy in the form of biomass and discussing the option for reusing non-hazardous medical waste, such as saline or glucose. Medical waste neutralisation during chemical disinfection, and subsequent disposal of the safer substances, is most likely to be an alternative to heat treatment, avoiding the use of incineration. However, incineration has provided energy but must be closely monitored and serviced accordingly. This will ensure safety and avoid incomplete burning, which leads to harmful emissions. A sustainable waste management system for medical waste must adhere to policies and legislation surrounding this topic along with effective segregation and treatment. There must also be regular collections, transportation and the appropriate storage and careful attention to treatment of medical waste. During segregation, recycles should be considered, such as glass vials and plastics. A system that is considerate of these activities has the possibility to work well and improve the reduction of medical waste by reducing the amount generated. Table 1 indicates the current technologies available that are used for disposal of healthcare waste including medical waste in the countries of Africa. This table identifies the risks associated with contamination, land, air, humans, wildlife and water and therefore proposes more economically viable solutions than the current methods.

## Discussion

### Findings of reviewed studies

The papers researched in this review have investigated the storage, disposal and transportation of medical waste in African countries. Of the selected papers, there are not many that have discussed both issues of medical waste and sustainable models for a circular economy; the reduce–reuse–recycle approach. The results shown in Table 1 have identified the more sustainable models for medical waste. The incineration process is applied in different countries, including Nigeria, Algeria, Botswana, Ethiopia and South Africa. Although it is used for disposal of infectious and hazardous wastes, but it results in a huge amount of ash and causes environmental pollution. Open dumping and uncontrolled landfills are used in countries, such as in South Africa, Nigeria and Botswana because they are cheap and easily available; however, they expose the public to health risks, as well as land and water contamination, and air pollution. In South Africa and Nigeria, autoclaving of wastes is applied. Even though it is cheaper than incineration and helps to get rid of the bacteria from contaminated wastes, it is difficult to treat a high quantity of wastes due to expenses of electrical usage. Chemical disinfection is used in Algeria, and it is useful for destruction the pathogenic substances; however, it has the risk of contact with chemicals, as well as possible contamination in case of insufficient treatment. The current technologies used are fossil fuel derived, electrical, or have the potential to be hazardous to health and the environment due to chemical exposure. These methods are not sustainable and put many workers and households in the surrounding communities at considerable risk. These reviewed studies and articles have also identified current active processes regarding disposal of healthcare waste in various African countries, cities and suburbs. It is apparent that although some hospitals are efficient in providing adequate healthcare waste facilities, this is not the case in many healthcare centres and hospitals, which need improvement.

These studies have suggested that there is a lack of understanding across the continent about the proper disposal methods of healthcare and medical waste, and therefore this leaves potential for significant risk and issues in the future. As Africa is going through socio-economic change and rapid industrialisation, a more sustainable method for medical waste should be introduced to move forward with a circular economy, and a reduce–reuse–recycle approach. This means the reduction of medical waste by reducing medical waste at the site where it originated, reuse items that can be reused in a safe manner, and recycle glass, plastics, paper, cardboards and other recyclable general waste. Healthcare facilities and pharmacies should implement changes that ensure responsibility across the board, from the place of origin to final disposal. Prohibiting improper disposal, such as open incineration and illegal indiscriminate waste disposal, has the possibility of reducing these issues. Education on how to create and manage biofuels to harness energy and the recycling of non-hazardous medical waste should be discussed to enable a more circular economy and contribute to the mitigation of climate change for all African countries. From the objectives of this paper, it is apparent that although some regions of some African countries can manage healthcare waste, there is still much to be discussed regarding medical waste. There is currently no active effective system to guide healthcare workers or pharmacists in rural and vulnerable parts of Africa on how to dispose safely and efficiently of medical waste. A transfer system needs to be implemented, with allocations of adequate transport resources per country/region and the agreement of collecting, storage and transporting to the final disposal in accordance with the WHO guidelines. In addition, implementing Ghana’s DUMP programme across the continent would be invaluable to the areas that do not have an available medicinal return facility. This programme would allow residents to dispose of their unused, expired and partially used medicines without irresponsible disposal, such as flushing or mixing with general waste. This would result in reduced accumulation in households across Africa and would lessen the risk of contaminated soil and water, as well as bringing a reduction in the release of APIs into the surrounding environment, with the potential to harm human beings, and wild and farmed animals. At the time of writing this paper, some countries remain in conflict, and being unanimous could see the growth of socio-economic well-being and tackle climate change issues.

An effective healthcare waste management system would be highly beneficial. Non-hazardous medical waste, such as saline or glucose can be recycled. Furthermore, in the 1960s, medicals were packaged into blister packs to prolong shelf life as opposed to glass bottles. The blister packs established an environment that was free from gases and moisture, as well as ensuring a packaging that would suggest whether tampering had occurred, with the additional benefit of being childproof. These packs also allowed for the user to keep track of medicine use (Nieminen et al., 2020). Usually it is a case that if medicines in a blister pack are unused or expired, the whole of the package is discarded into general waste, but due to its components of aluminium and plastic layers, it is available for recycling (Nieminen et al., 2020). This has increased the opportunity to lead to a more circular economy approach by means of the reduce–reuse–recycle process and thus contribute to a sustainable future. To further establish sustainability, a multitude of other sectors must be considered; firstly, the environment. In Africa, an environmental impact assessment (EIA) is required before any project or development is decided for the economy. This assessment will consider culture, human health and the socio-economic impact (Omwoma et al., 2017). This policy was published by the US National Environmental Policy Act of 1969 (Grossarth and Hecht, 2007). Countries in Africa, such as Kenya, Zambia, Senegal, Algeria, Togo, Gabon, Burkina Faso, Gambia, Mauritius and Nigeria have followed this EIA process as a requirement for any development proposal since 1980 (Betey and Essel, 2013). Another factor to consider for a sustainable economy is energy consumption. Many households in Africa still live without electricity and are dependent on fossil fuels for cooking and heating, with many going without electrical lighting. Using fossil fuel energy products induces toxic emissions and greenhouse gases into the atmosphere, something that is being combatted around the world. According to the Global Burden of Disease study, around 4 million people die every year of illnesses caused by household cooking fuels (Pachauri et al., 2013). As the concern of fossil fuel derived energy has been the main cause of harmful emissions and greenhouse gases, the issues have been raised within economies, leading countries to tackle and mitigate climate change with sustainable agendas (Gautam et al., 2020; Le and Van, 2020; Rubaai and Young, 2011).There are plentiful other types of renewable energy resource to be considered. Harnessing energy from sustainable sources such as wind, geothermal, solar, hydropower and biomass is admirable and can contribute to mitigating climate change and encouraging sustainable development (Le and Van, 2020). Biomass waste is considered an attractive source of energy that includes a high variety of different fuels. The high biomass share in the energy consumption has been found in various developing countries, where the biomass are mainly used for cooking and heating. Approximately half of the energy is used mainly from biomass as well as agricultural residues in Africa, which is projected to be the highest in the world for bioenergy production from the biomass by 2050 (Gautam et al., 2020).

### Approaches and suggestions

Developing countries are trying to develop new efforts for more comprehensive schemes regarding eco-friendly medical waste management. However, countries should not directly implement the approaches used by others, and they must learn from forerunners and develop a proper waste management plan according to the country’s perspective (Mmereki et al., 2017). The management plan implemented should take into account the political, fiscal, scientific, technical, social and economic aspects (Oke, 2008). Some suggestions and approaches could be introduced to face the fiscal, economic, social and political barriers characteristic of developing city problems as following: prescribing the minimum required amount of medicines per person during a clinic visit, establishing national level policies via the governments as well as including them in the legislation is important to achieve effective and successful programmes in the developing countries; international organisations, such as WHO, should participate their experience and supply adequate funds to achieve successful programmes of sustainable medical waste management in the developing countries; collaboration of multiple governmental and non-governmental institutions can help in achieving successful programmes for waste management; performing pilot projects can help designing and establishing programmes that can meet the local needs; effective educational programmes should be presented to the stakeholders, such as doctors, pharmacists and consumers; establishing guidelines and publishing informative websites, which include data and information about the proper disposal of medical wastes; continuous and multi-level education is essential to increase the knowledge as well as the right attitude of stakeholders; the unused medicines can be expired or non-expired; the expired medicines should be incinerated by municipal authorities, whereas non-expired medicines can be returned by offering discounts for the consumer purchases or encouraging the public by making based initiatives, such as ‘National take back day for medicines’; surveys of periodic feedback and quality assurance monitoring can help in understanding the current problems and improving the systems on a regular basis, developing countries should join the international education programmes and forum on medical waste management to start achieving successful programmes of medical waste management in their countries (Bataduwaarachchi et al., 2018); it is essential to educate and inform the stakeholders, such as hospital staff, waste handlers, patients, visitors and the general public, the negative impacts and health risks of improper disposal, handling and management of hospital wastes (Ali et al., 2017); and it is essential also to know that some developed countries are still facing challenges to make the behaviour and actions of hospital employees and stakeholders more sustainable (Tudor et al., 2007).

### Strategies and action plans

The WHO recommended that the choice of the mode of treatment and disposal of medical waste should be guided by cost-effectiveness, easy implementation and environmental friendliness (Hossain et al., 2011). Moreover, the countries should work to establish sustainable waste management chains, including addressing logistics, recycling, treatment technologies and policies (WHO, 2020). Collaboration between different governmental institutions could achieve effective programmes for waste management. The institutions can be collaborated with the Ministry of Health, Ministry of Environmental Protection, municipal councils water board, legal departments, law enforcement authorities, hospital and pharmacy network, universities and consumer authority. The Ministry of Health should hold the leading role. A multidisciplinary committee should help in drafting policies and be responsible for the overall supervision of the programmes. Universities and other academic institutions have the responsibility of providing expert opinions on medication waste management. Integration of this subject with undergraduate curricula will improve the understanding among junior doctors and pharmacists about the programmes as well as their responsibilities. Establishment of post graduate programmes, such as ecotoxicology and pharmacovigilance, will produce local experts in the subject. It is always good if the principles of medication waste management can be included in school curricula as well (Bataduwaarachchi et al., 2018).

On the other hand, proper medical waste management can be achieved by the following actions: training and educating the hospital staff how to achieve the efficient and safe waste management, as well as informing them how the avoidance and minimisation of wastes can decrease work burden on the staff; realising the staff that they are the primary stakeholders and they have important role to create of a clean and hygienic work environment; vaccinating the working staff against infectious diseases and relocating infected staff away from the patient wards (Raggam et al., 2009); emphasising during the training that the segregation of wastes should be performed using PPE (Makajic-Nikoli et al., 2016); training paramedic staff, especially the nurses, to avoid spillage or mixing of wastes, as well as separating non-risk wastes, such as food wastes, paper and plastic bottles that can be easily and safely treated or recycled (Unger and Landis, 2016).

To establish sustainable medical waste management chains, strategies and action plans should be undertaken. The hospital waste should be segregated into colour-coded and labelled bags or containers (Marinković et al., 2008). Proper segregation can reduce the risk of diseases, such as hepatitis and human immunodeficiency virus infections (Prüss-Üstün et al., 2005). Moreover, source segregation can also help reduce the fraction of waste required to be incinerated, thus conserving energy and save money (Alvim-Ferraz and Afonso, 2005). Rules regarding waste storage generally require the waste to be stored temporarily in properly labelled separate store rooms (Ali et al., 2017). The storage areas need to be well ventilated with water and sewerage access. These locations should be properly labelled with warning signs and should have restricted access limited to the workers only and inaccessible to rodents and unauthorised people for a maximum of 48 hours and then transported to the treatment or disposal site (Olaniyi et al., 2018). Medical waste transportation should be regulated by using an online tracking system to monitor medical waste transportation, where information regarding waste characterisation, generator, transporter and the treatment facility is duly recorded (Jang et al., 2006). In addition, a licensed transporter should be allowed to transport the waste (Mmereki et al., 2017).

The treatment and disposal methods of medical wastes should be feasible, economic and eco-friendly and sustainable methods (Hossain et al., 2011). Open dumping, because of its inherent problems, such as leakage of toxic substances into the environment; easily accessed by insects, rodents and the small animals, most of which are disease vectors, has been replaced by engineered sanitary landfills in the modern waste management to minimise the risk assessment of the landfill hazards to preserve the environment and human health. Moreover, landfill system should be located at a distance away from the healthcare facilities, and it must be properly designed, operated and monitored in accordance with permit conditions (Olaniyi et al., 2018).

The incinerators should be provided with emission control systems and fitted with typical air pollution control devices, including cyclones, semi-dry scrubbers and baghouse filters. Moreover, the incinerator should have tall chimney to emit gases far from communities and avoid nuisance and potential causes of bronchitis and pulmonary ailments, such as asthma (Al-Khatib and Sato, 2009). Properly designed incinerators should completely burn waste leaving a minimum of residuals in the form of ashes. Regular monitoring of the emissions from the incinerators would generate the necessary data, which could enable the governments to accurately estimate the impact of medical waste on the environment (Mmereki et al., 2017).

Environmentally friendly alternative treatment technologies for medical waste, including microwave sanitation, chemical disinfection, dry heat disinfection, disinfection with superheated steam, gasification, pyrolysis and anaerobic digestion can be applied (Ali et al., 2017; Mmereki et al., 2017). Gasification and pyrolysis are novel and eco-friendly techniques used as thermochemical treatment of wastes as well as energy recovery. Plasma gasification and pyrolysis represent a state-of-the-art solution for sustainable management of medical. A plasma gasifier is an oxygen-starved reactor that is operated at the very high temperatures, which result in the breakdown of wastes into hydrogen, carbon monoxide, water, etc. The main product of a plasma gasification plant is energy-rich syngas, which can be converted into heat, electricity and liquid fuels. Inorganic components in medical wastes, like metals and glass, get converted into a glassy aggregate (Zafar, 2019). Pyrolysis is a thermal treatment of medical wastes in the absence of oxygen, which can produce gaseous and liquid products that can be used as a source of chemicals and fuels (Som et al., 2018). Anaerobic digestion is an environmentally friendly technique used for biochemical treatment of wastes and produce biogas that can be used for heat and power generation (Abdel Daiem et al., 2018; Said et al., 2020). Furthermore, renewable energies from solar, wind and biomass can also meet the energy requirements of waste treatment technique and mitigate greenhouse gas emissions generated from fossil fuel consumption for energy generation (Said et al., 2020). This can support decision-makers in developing strategies for the sustainability by using the eco-friendly and economic technologies for efficient medical waste treatment that can be implemented in the developing countries.

### Alternatives of treatment and disposal methods

Categories of healthcare waste according to WHO as well as waste disposal alternatives are summarised in Table 2. Risk wastes including infected wastes, infected sharps and genotoxic waste must be incinerated. Otherwise, non-risk wastes, such as paper, plastic, cardboard and packaging could be treated using eco-friendly techniques, such as anaerobic digestion and pyrolysis, which could produce clean and renewable energy (Ahmed et al., 2019; Ali et al., 2017; Said et al., 2013). Sharp items consisting of needles, syringes and broken glass can be steam sterilised and subsequently recycled or treated using plasma gasification technique, as well as energy recovery (Zafar, 2019). Medical waste, such as expired or unused medical products, surplus drugs, vaccines or sera can be periodically draining in limited quantities while discarded items used in handling medical waste, such as bottles, boxes, gloves, masks, tubes or vials, can be returned to pharmacies or manufacturer and recycled (Ali et al., 2017). Chemical wastes usually consist of items from diagnostic work, housekeeping procedures and discarded batteries. These items can either be treated onsite through dilution or collected by original equipment manufacturers or recyclers (Kookana et al., 2014). Radioactive waste includes items contaminated with radionuclides. These items should be stored in lead containers and collected by appropriate government agency for treatments using deep underground burial or membrane technology (Sancho et al., 2013). Categories of healthcare, constituents and alternatives of waste treatment and disposal methods can serve as a link between the healthcare system, decision-makers, and stakeholders in developing health policies and programmes. These findings consistence with United Nations Sustainable Development Goals for sustainability and waste management that included protection of public health and environment for achieving sustainable cities, good health and clean energy to compact climate change. Control and proper waste management to prevent air, land and water pollution, as well as using clean and renewable energy to prevent greenhouse gas emissions represented the specific targets to minimise the adverse impacts on human health and the environment (UN, 2016).

**Table 2.** Categories of healthcare wastes according to WHO, and treatment and disposal options.

Waste category	Constituents	Treatment and disposal options
Risk waste	Infectious waste, infected sharps, and genotoxic waste	Incineration
Non-risk waste	Paper, plastic, cardboard and packaging	Anaerobic digestion – Pyrolysis
Sharps	Needles, syringes, scalpels, infusion sets, saws and knives, blades and broken glass	Autoclaving/microwave treatment and recycling
		Plasma gasification
Pharmaceutical waste	Expired or unused pharmaceutical products, surplus drugs, vaccines or sera and discarded items used in handling pharmaceutical waste, such as bottles, boxes, gloves, masks, tubes or vials	Returned to pharmacies or manufacturer
		Recycling
		Periodically draining in limited quantities
Chemical waste	Chemicals from diagnostic and experimental work, cleaning processes, housekeeping and disinfecting procedures, and discarded batteries	Chemicals dilution – batteries recycling
Radioactive waste	Liquid, solid and gaseous waste contaminated with radionuclides generated from in-vitro analysis of body tissue and fluid, in vivo body organ imaging and tumour localisation, and investigation and therapeutic procedures	Treating and burying by appropriate Government agency

## Conclusion

Improper handling of medical waste has been shown to have debilitating effects on the environment, including wildlife, water quality and the large risk of spreading disease. As well as the cost of disposal of healthcare waste, factors, such as contamination and pollution, must be considered when this type of waste is being stored and transported, and decisions are being made on the correct technology for disposal. We have shown above examples of countries in Africa that use a multitude of disposal technologies and economic solutions. Colour coding and recorded seals have proven to be an efficient process, and they follow the protocol of many legislations and policies that prevent the negative impact of healthcare waste on the environment. As there is certainly a gap for discussion of a sustainable waste management system to provide a more circular economy within the whole of the African continent, this paper has recognised some of the leading issues in modern-day Africa. In conclusion, and in recognition of the proposed concerns, Africa has the full potential for thriving economic changes. However, this requires investment from both government and international funds and sustainable energy sources, with the addition of extensive waste management, innovative technologies, and implementation of political enforcement. Green programmes and unanimity across Africa would build a flourishing future. It is apparent that controlling waste by means of reduced production would be a suitable conclusion, as well as ensuring only hazardous waste is accumulated for treatment, and all other healthcare waste is treated accordingly. In addition, separating matter, such as food waste and recyclables from regular hospital waste, would also contribute to efficient waste management by allowing the possibility of using biomass from food waste and recycling paper/cardboard. Medical blister packaging being correctly recycled could give rise to further sustainability. Despite many parts of the continent are still severely deprived owing to the shortage of clean water, poor education, restricted electrical resources and indiscriminate waste, implementing a universal programme to suit all countries across the continent has the potential to significantly reduce negative impact to the environment.

### Limitations

The limitation of this review is that it is focusing on studies related to the sustainability aspects of medical waste management in developing countries of Africa to present resilient solutions for health and environment protection. However, there is a need to review more studies for developed and developing countries of Africa to make sufficient comparison for the current situations in countries and discuss sustainable models for a circular economy, as well as research sustainable and environment friendly solutions for medical waste disposal in more detail.
